# Adaptive Optics of Kyrieleis Plaques in Varicella Zoster Virus-Associated Posterior Uveitis: A Multimodal Imaging Analysis

**DOI:** 10.3390/jcm12030884

**Published:** 2023-01-22

**Authors:** Paolo Milella, Chiara Mapelli, Marco Nassisi, Gaia Leone, Giada Ruggi, Antonio Scialdone, Giuseppe Casalino, Francesco Viola

**Affiliations:** 1 Department of Clinical Sciences and Community Health, Università degli Studi di Milano, 20122 Milan, Italy; 2 Fondazione IRCCS Ca’ Granda Ospedale Maggiore Policlinico di Milano, 20122 Milan, Italy; 3 Ospedale Fatebenefratelli e Oftalmico, ASST Fatebenefratelli Sacco, 20121 Milan, Italy

**Keywords:** Varicella Zoster virus, Kyrieleis plaques, adaptive optics, multimodal imaging, uveitis

## Abstract

Kyrieleis plaques (KP) represent a peculiar type of vasculitis affecting retinal arterial branches in a beaded segmental pattern that can be found in several posterior inflammatory ocular conditions. The nature and precise location of KP is unclear. Adaptive Optics (AO) provides an in vivo visualization of retinal vasculature on a microscopic level, thus permitting a more detailed characterization of KP as compared to traditional imaging techniques. This study aims to report AO imaging of KP in Varicella Zoster virus (VZV)-associated posterior uveitis and to correlate the findings with traditional imaging techniques. Three patients diagnosed with VZV posterior uveitis underwent adaptive optics (AO) imaging and traditional multimodal imaging techniques, including fundus photography, fluorescein angiography, indocyanine green angiography and optical coherence tomography. In all subjects, AO imaging revealed segmental hyporeflectivity confined to the vessel wall, with no evidence of arterial wall disruption or extravascular involvement. In our series, AO findings support the view that KP are localized within the inner arterial wall, possibly at the endothelial level.

## 1. Introduction

Kyrieleis plaques (KP) are a non-specific retinal finding that reflect severe intraocular inflammation [1,2]. They appear as white-yellowish exudates scattered in a beaded, segmental pattern within the retinal arteries and they have been reported in association with several conditions such as Toxoplasma Gondii retinochoroiditis, Mycobacterium Tuberculosis, Rickettsia Conorii, Treponema Pallidum, Herpes virus type 1 and type 2 (HSV-1, HSV-2), cytomegalovirus (CMV) and Varicella Zoster virus (VZV) infections [2,3,4,5,6,7,8].

VZV uveitis can affect any segment of the eye. It can manifest with sight-threatening alterations including vitritis, retinal hemorrhages, vasculitis, retinitis and retinal necrosis [9,10,11]. While the diagnosis is based on the typical clinical and imaging findings, a polymerase-chain reaction (PCR) testing is often advised to confirm it. Retinal vasculitis in these cases typically affects arteries [12] and may lead to vascular occlusion and/or rupture of the blood-retinal barrier resulting in retinal hemorrhages and exudation. Arterial vasculitis can manifest in the form of localized arteritic plaques, namely KP.

The etiology of KP is unclear, as is their composition and exact location [2]. It has been hypothesized that they might represent deposition of inflammatory cells and debris, arteriosclerosis, or exudates migrated from underlying retinochoroiditis foci. Although classically referred to as segmental retinal ‘periarteritis’, a recent study using multimodal retinal imaging showed that KP affect the artery wall, thus speculating a primary role of the inflamed endothelium in the pathogenesis of these findings [13]. However, there is no pathologic study assessing these lesions on histologic scale.

Adaptive optics (AO) is a novel non-invasive, high-resolution imaging modality that enables in vivo visualization of retinal microstructures on a quasi-histologic scale. This technology was originally used in astronomy to overcome light aberrations when capturing light from outer space. This principle has later been applied to ocular imaging to overcome high-grade aberrations of the eye’s dioptric means, in order to obtain high-resolution images of the retina. The use of AO has already provided substantial pathologic insights in different retinal diseases [14,15], since it allows for precise visualization of retinal microstructures such as photoreceptors and blood vessels. Therefore, AO offers an invaluable opportunity to study KP in detail.

The aim of our study is to characterize KP with AO imaging and to define the relationship of these findings with those of the traditional imaging techniques.

## 2. Materials and Methods

Retrospective case-series of patients with a diagnosis of KP in VZV-associated uveitis seen at Fondazione IRCCS Ca’ Granda Ospedale Maggiore Policlinico di Milano, Milan, Italy and at Ospedale Fatebenefratelli e Oftalmico, ASST Fatebenfratelli Sacco, Milan, Italy, from May 2017 to July 2017. This study used data from electronic medical care records and associated imaging repositories of patients seen in two tertiary referral eye care centers. 

The patients underwent comprehensive ocular examination including best-corrected visual acuity, slit-lamp examination and multimodal retinal imaging: color fundus photography (FP—Canon CR1 Mark II; Canon Inc, Tokyo, Japan), fluorescein angiography (FA—Heidelberg Spectralis; Heidelberg Engineering, Heidelberg, Germany), indocyanine green angiography (ICGA—Heidelberg Spectralis; Heidelberg Engineering, Heidelberg, Germany), spectral-domain optical coherence tomography (SD-OCT—Heidelberg Spectralis; Heidelberg Engineering, Heidelberg, Germany) and AO flood illumination ophthalmoscopy (rtx1-e AO retinal camera; Imagine Eyes, Orsay, France). 

A presumptive diagnosis of herpetic vasculitis was made on the basis of presenting clinical features (i.e., vitritis, vasculitis, more rarely papillitis), and treatment was promptly started according to recognized guidelines [16]. Then, VZV etiology was confirmed in all patients through quantitative real-time PCR (RT-PCR) analysis on aqueous humor tap using the VZV ELITe MGB^®^ Kit (ELITechGroup, Italy). 

All imaging was performed after treatment initiation, as soon as the vitritis cleared enough to allow a proper visualization of the fundus. Once KP were identified with clinical examination, retinal areas of interest were localized with FP and then assessed with the other imaging techniques. Angiographic images were obtained in early and late phases. Four-degree field AO images were taken on the regions of interest with a standard acquisition protocol with the rtx-1 in-built software. Several images were taken for each region of interest and the best quality image was then selected for analysis. Multimodal imaging of involved arterial segments was then analyzed. 

## 3. Results

A total of five patients presented with VZV-associated posterior uveitis in the study period; three of them showed KP at fundus examination and were included in the study. Two males (61 and 78-year-old) and one female (79-year-old) were included. Imaging was performed between 2 weeks and 2 months after presentation, depending on ocular means clarification and availability of the patient. 

In all subjects FP showed whitish, segmented deposits along retinal arteries, which did not extend beyond the artery wall, with no venous involvement. FA and ICGA images showed iso-hypofluorescent lesions corresponding to the deposits, without vascular leakage or staining in the early and late phases. OCT scans passing through the lesion showed a hyperreflective band at the level of the affected arterial wall. In all subjects, AO imaging revealed hyporeflective lesions confined to the vessel wall, with no evidence of arterial wall disruption and extravascular involvement. Hyperreflective glistening spots were also detected on the longitudinal axis of the affected segments of the arteries. The axial extent of the lesions was variable among the cases and had a segmental pattern in all of them. 

**Figure 1:**
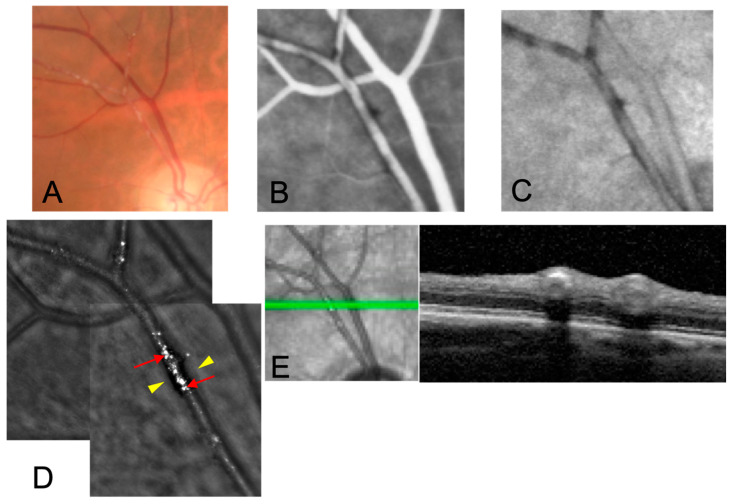
Multimodal imaging of case 1. A 78-yo man under steroid therapy for rheumatic polymyalgia developed VZV uveitis with arteritis and vitritis. RT-PCR confirmed the presence of viral genome in the aqueous humor (7,658,000 genomes/mL). He was treated with oral valacyclovir 2 g three times daily with good clinical improvement. (**A**) KP appear as whitish segmented deposits within the artery wall on FP; (**B**) late phase FA and (**C**) ICGA shows no leakage nor staining of the lesions; (**D**) AO imaging shows segmental arterial hyporeflectivity (yellow arrowheads) and adjacent granular hyperreflective spots on the vessel longitudinal axis (red arrows); (**E**) OCT scan shows hyperreflectivity of the arterial wall with underlying hyporeflective signal.

**Figure 2:**
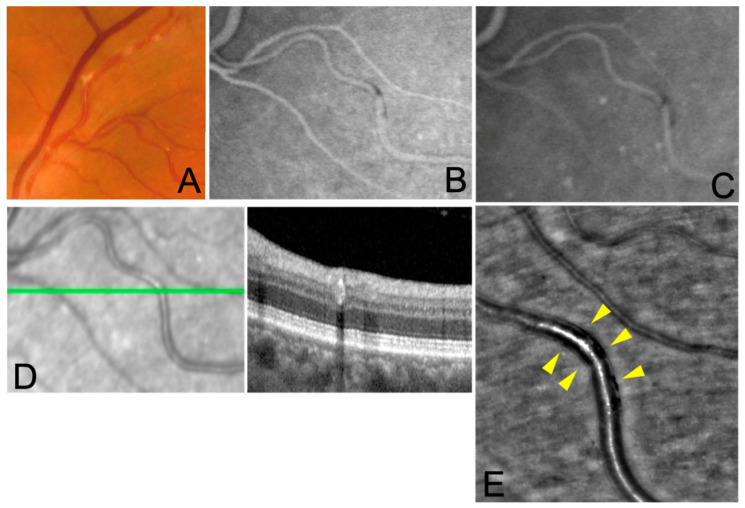
Multimodal imaging of case 2. A 61-yo healthy man was diagnosed with VZV posterior uveitis confirmed through RT-PCR analysis on aqueous tap (2,292,000 genomes/mL) and was then treated with oral valacyclovir 2 g three times daily and corticosteroids. (**A**) KP can be detected along retinal arteries on FP, of note the venous vasculature is spared; (**B**,**C**) KP appear as hypo/iso fluorescent lesions in both early and late phase in FA and ICGA; (**D**) focal hyperreflectivity of the arterial wall on OCT with underlying hyporeflective signal; (**E**) segmental hyporeflectivity (yellow arrowheads) is clearly visible within the artery wall.

**Figure 3:**
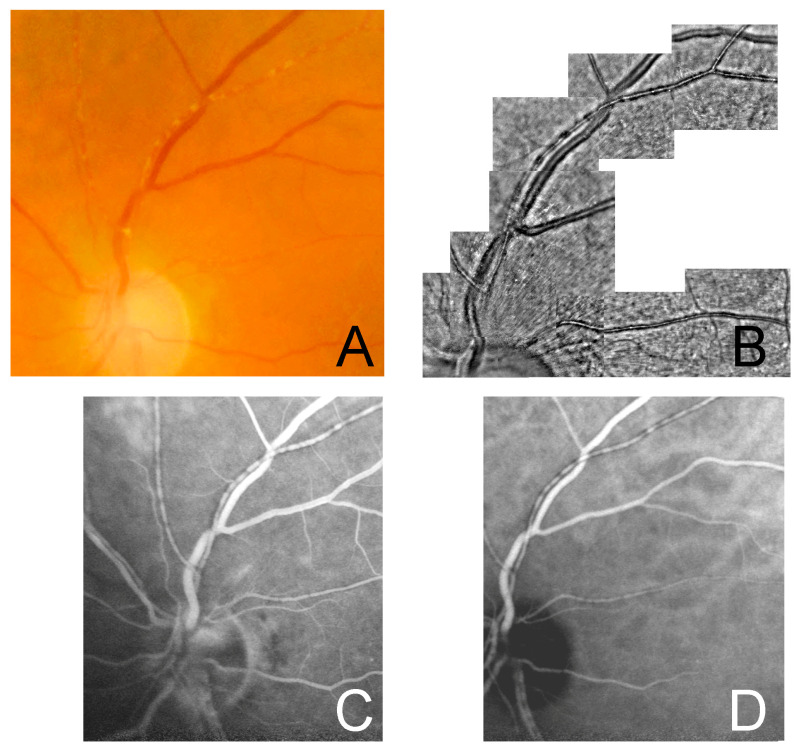
Multimodal imaging of case 3. A 79-yo female with VZV-related acute retinal necrosis confirmed with RT-PCR analysis on aqueous tap (1,255,340 genomes/mL), imaged two months after treatment with intravenous acyclovir 10 mg/kg three times daily and subsequent oral valacyclovir 1 g three times daily. (**A**) FP showing wide multifocal arterial involvement with a segmented pattern; (**B**) AO confirms the presence of KP as segmental hyporeflectivity in multiple sectors of the arterial vascular branches; (**C**,**D**) late phase hypofluorescent vascular lesions are seen on FA and ICGA.

Multimodal imaging of the three cases is shown in Figure 1, Figure 2 and Figure 3. 

## 4. Discussion

The present study investigated the role of AO in elucidating the composition and location of KP in Varicella Zoster virus (VZV)-associated posterior uveitis.

The pathophysiology of KP is indeed unclear. Previous studies suggest that they may be inflammatory infiltrates that are limited to the arterial vessels wall [3,5,17]. Alternative hypotheses are that KP may be calcific deposits in the arterial walls or the result of migration of exudates from the active choroiditis to the periarterial sheaths [1,2,18].

Recently Pichi and coauthors described these lesions using multimodal imaging including FA and ICGA [13] and hypothesized a primary pathogenetic role of the inflamed endothelial cells in the retinal arterioles. While a histologic exam might be revealing about the intrinsic nature of these lesions, it is, however, unfeasible. 

AO, with its quasi-histologic resolution, can be a useful alternative for the in vivo assessment of these lesions. AO imaging has been widely used to obtain high-resolution images of retinal vasculature in uveitis, showing signs like perivenular sheathing or vasculitis in subclinical stages (i.e., not visible in other imaging modalities) [14,15,19]. To the best of our knowledge, this is the first study reporting use of AO in KP.

In all three cases in our study AO showed a segmental hyporeflectivity confined to the vessel wall. This finding does not seem to be consistent with the initial hypothesis of the involvement of the periarterial sheath [20]. By contrast, our findings are in line with the observations made by a recent in vivo study [13] that has suggested the involvement of the vessels’ endothelium. We therefore contend that the use of the term ‘periarteritis’ referring to KP should be discouraged. 

Of note, in correspondence of the KP, on AO we also observed adjacent granular hyperreflective spots on the vessel’s longitudinal axis. The nature of this finding is unclear and may represent migration of inflammatory cells from the primary site of inflammation. 

Unlike the previous reports, we did not find any hyperfluorescence signal on ICGA in correspondence of the lesions. This may be explained by the acquisition of the images in the due course of the disease, after treatment initiation, thus reflecting a sub-acute resolving phase with scarce activity of the lesions. This explanation is consistent with the observation that in the minority of the cases in which a glistening appearance of the vessels persisted after treatment, ICGA showed hypofluorescence. 

In our study, in agreement with the findings of previous studies [2,13], OCT of KP showed focal hyperreflectivity of the vascular wall and correspondent hypofluorescent lesions on ICGA. No outer retinal abnormalities were detected on OCT in the adjacent area. These tomographic findings may support the hypothesis that KP derives from a primary affection of the endothelial cells that, when damaged, lead to exudation and deposition of fibrin and other inflammatory molecules within the arterial wall. Once organized, they form plaques that may not be easily degraded or cleared by the immune system. This mechanism may explain the correlation between the hypofluorescent appearance in ICGA and FA (from masking effect) and the hyperreflectivity in OCT. Of note, it would be of interest to test our hypothesis by performing a confocal microscopy study on cultured endothelial cells and assess their deposition process.

The present study has several limitations; the major limitations are represented by the small cohort analyzed, and the lack of a control group. It is also noteworthy to acknowledge that because of the cross-sectional nature of the study we may not exclude the fact that AO may have shown different findings at different stages and severity of the disease.

Furthermore, the use of AO imaging has some limitations in daily clinical practice. Firstly, it needs clear ocular means as it is unable to image the retina in presence of vitritis, cataracts, severe anterior chamber reaction or corneal scarring. Secondly, it only allows visualization of the central retina, ranging about 25–30° from the posterior pole: a proper study of peripheral retinal conditions (as usually is vasculitis, among others) may be impossible. Moreover, the acquisition process and the consequent image quality are dependent on the patient’s compliance, as a good fixation is needed. Lastly, the cost of this imaging technique in terms of time and resources, as well as its relatively low availability in ophthalmology clinics, may represent limitations to its clinical use. 

## 5. Conclusions

In conclusion, in this study all imaging modalities allowed visualization of the KP but AO made it possible to localize KP lesions in greater detail, suggesting a higher sensibility in detecting microvascular changes compared to traditional imaging techniques. In our series, AO findings support the view that KP location is within the arterial wall, possibly at the endothelium level. Further studies are needed to confirm our findings. 

## Data Availability

The raw data supporting the conclusions of this article will be made available by the authors, without undue reservation.
